# Social determinants of self-medication practice by caregivers among pediatrics population in Pakistan: a logistic regression analysis of a prospective cross-sectional study

**DOI:** 10.1097/MS9.0000000000003947

**Published:** 2025-10-06

**Authors:** Abdul Ghani, Usha Kumari, Uzair Yaqoob, Zair Hassan, Vu Thi Thu Trang, Shanzay Zahid, Aarash Khan

**Affiliations:** a Jam Ghulam Qadir Government Teaching Hospital Hub, Quetta, Pakistan; b Dow University of Health Sciences, Karachi, Pakistan; c Hamdard Teaching Hospital, Karachi, Pakistan; d Lady Reading Hospital, Peshawar, Pakistan; e National Hospital of Traditional Medicine, HaNoi, VietNam; f Herat University, Herat, Afghanistan

**Keywords:** caregivers, caretaker, children, literacy rate, OTC drugs, over-the-counter drugs, Pakistan, pediatrics, self-medication

## Abstract

**Background:**

Self-medication (SM) is described as the usage of medication without authorization or prescription from a certified physician. SM in children is a rising concern, especially in developing countries like Pakistan. This study aims to determine the frequency of SM in children under 5 years (SMC5), practiced by their caregivers (CGs), and the factors leading to it.

**Materials and methodology:**

From August to September 2021, a single-centered cross-sectional survey was conducted using a standardized questionnaire administered by an interviewer. CGs provided informed consent before participating in the study. Statistical analysis was carried out with IBM Statistical Package for the Social Sciences (SPSS) version 23.0.

**Results:**

Our study comprised 476 final participants. Approximately half (45.8%) of the children were in the age group of 1–3 years, 36.1% were under 12 months old, and 16.8% were between 3 and 5 years old. The majority of the CGs were women (96.01%). 53.99% of CGs did not have primary education indicating a very low literacy rate. A vast majority (83.1%) of CGs had drugs available at home with 42.9% of the population getting them from a pharmacy. 94.8% of the CGs had very poor knowledge regarding the correct timing, dosage, and adverse effects of the drug, resulting in irrational use of the drugs.

**Conclusion:**

According to this study, SMC5 is a common practice that carries significant health risks. Governments, healthcare authorities, and educational institutions can work together to address this issue.

## Introduction

Self-medication (SM) refers to the use of over-the-counter (OTC) or unprescribed medications by the patient or caregiver (CG), for prevention, diagnosis, improvement, or treatment of any medical illness, without consulting a physician or other qualified healthcare provider. It also involves arbitrary changes in the dose of medicines without a doctor’s advice^[1]^. SM is a hot topic in both developed and developing countries, and it is one of the leading causes of mortality^[2–4]^. The prevalence of SM in developing countries varies from 12.7 to 95% in contrast to Western countries, where it is as low as 3%^[3]^. World Health Organization (WHO) reports that SM increases due to easy access to medication, increased accessibility of medical products, increased doctors’ advisory fees, commercialization, and online availability of pharmaceutical products^[5,6]^. According to a survey in Pakistan, around 96% of community pharmacy stores contribute to SM by dispensing medicines, including antibiotics, without a prescription^[7]^. It is increasing among educated individuals compared to illiterate individuals and is also common among healthcare workers^[8]^ and students^[9–11]^.

SM in children is found to have more lethal consequences due to medication errors^[4]^. Drug dosage and route of administration in children are based on their age, surface area, and weight; thus, improper use or a misloaded dose can be potentially toxic. With antibiotics, it may cause resistance^[9,12]^, while drug poisoning may occur at the wrong doses^[4]^. CGs, including mothers, unknowingly put the health of children in danger by giving them OTC drugs. This may be due to poverty, insufficient family support, and ease of access to medicines.

SM in the pediatric population is an overgrowing concern, and the literature is still deficient. Therefore, the current study aims to determine the extent and frequency, patterns, types of medications used, and risk factors contributing to SM in children under age 5 (SMC5).

## Materials and methodology

This community-based cross-sectional study was conducted from August to September 2021, among the population of Awaran, which is the fourth largest district in the province of Baluchistan, Pakistan. Its population is 122 011 as per the last census^[13]^. According to the records of 2012–2013, only 42% of the population is literate, with the majority being males^[14]^. The total healthcare facilities in the entire district include 1 District Health Quarter (DHQ) hospital, 15 Civil Dispensaries (CDs), 7 Basic Health Care Units (BHUs), 1 Maternal and Newborn Child Health (MNCH) center, 1 Tuberculosis (TB) Clinic, 5 Rural Health Centers (RHCs), and 1 mobile dispensary. In addition, only 19 doctors, 4 nurses, and 41 paramedic staff are available for the entire community^[14]^.

Ethical approval was obtained for this study under reference number 616/LRH/MTI. The estimated sample size calculated using the WHO sample size calculator was 385. The calculation was performed assuming a *P*-value of <0.05 and a 95% confidence level^[15]^. A total of 550 samples were considered in the defined duration, out of which 476 (86.5%) samples were utilized for analysis after scrutiny.

All the CGs who used medication for their child without a prescription were selected for the current study. The exclusion criteria were parents’ or CGs’ refusal to consent and whether the participant was a medical professional (registered nurse, registered medical practitioner, pharmacist, etc.).

Data was collected through an interviewer-administered structured questionnaire written in the Urdu language. Informed consent was obtained from each respondent, and trained lady health workers asked questions to the CGs about SM to their children in the preceding 3 months by the recall method. A pilot test, i.e., content validity index, was carried out to evaluate the validity of the questionnaire. Statistical analysis was performed using IBM SPSS version 23.0. Frequency and percentages were calculated for categorical variables, while means with standard deviations were calculated for continuous or numerical variables. The χ² test was used after stratification according to different variables to see the pattern of SM according to different variables.

## Results

Of the total 476 participants, 266 (56.0%) were males and 209 (44.0%) were females. The majority of the children were between the ages of 1–3 years (*n* = 217, 45.6%), followed by those under 12 months (*n* = 179, 36.1%), and 3–5 years (*n* = 80, 16.8%). The proportion of female CGs, including mother and grandmother, was much higher (96.01%) than that of male CGs (3.99%). The age of the majority of the CGs was less than 35 years. Illiteracy was high among the CGs, as 53.99% of them had not attended any school, and only 29.20 and 16.81% had gone to school and university, respectively. Households having a monthly income in the range of 20 000–50 000 Pakistani rupee (PKR) were 46.64%, while only 8.8% had the highest income, which was >50 000 PKR. Most of the households (56.3%) comprised an extended type of family having more than seven members. The rest of the socio-demographic characteristics are summarized in Supplementary Digital Content Table 1, available at: http://links.lww.com/MS9/A962.

**Figure 1.**
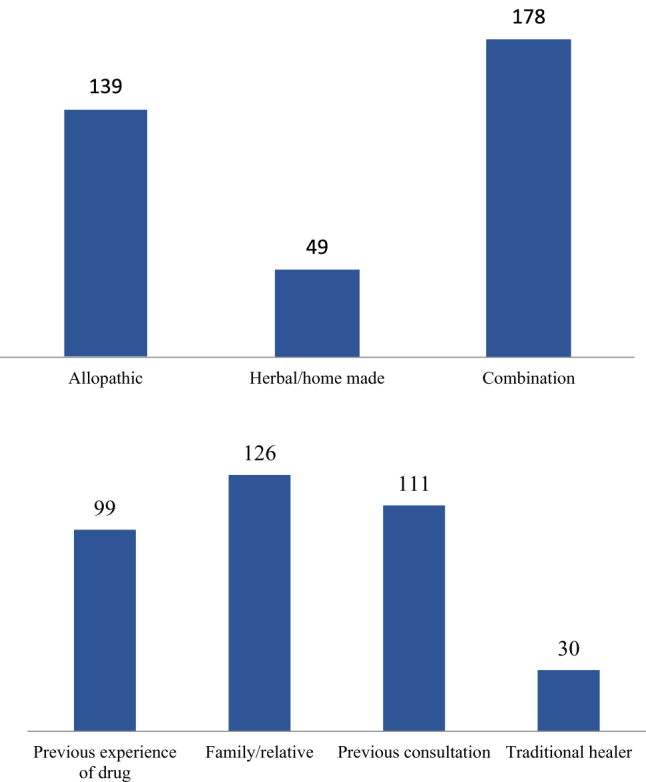
(a) Illustrates the type of drug used as a source of self-medication, and (b) shows the source of SM and its frequency.

A significant number (83.1%) of CGs had drugs available at home, the most common (42.9%) source of which was a pharmacy. 62.6% of the CGs started SM within 1–7 days after the onset of the illness, 49.7% continued it for 3–5 days, 51.4% discontinued it when the symptoms were not improving, and 46.1% discontinued it upon relief from the illness. A large percentage (95.1%) of the CGs started SM because they presumed the illness to be mild, with fever accounting for the majority of cases (71.3%), reinforced by the finding that most of the drugs available at home consisted of antipyretics/non-steroidal anti-inflammatory drugs (NSAIDs) (65.3%). However, there is not much variation in the availability of antipyretics, analgesics, and antibiotics at home, with 64.2% analgesics and 62.8% antibiotics at home. When asked about the methods of treatment in the last 3 months, 110 (23.1%) CGs reported having consulted a doctor and used only the prescribed medications, 198 (41.6%) had self-medicated, while 168 (35.3%) did both. Irrational use of drugs was common among the majority, as 94.8% of the CGs had no clue about the timing and dosage of the drugs, 94.3% did not know if the drug they were using could be given to a child or not, 83.6% did not read information available on the leaflets before using it, 76.2% did not have any idea about the potentially dangerous side-effects of the drug, and 59.6% did not check the expiry date. Moreover, 61.7% of the CGs recommended and shared the drugs with others too, promoting the practice of SMC5 (Fig. 1). The rest of the details about patterns of SMC5 are given in Supplementary Digital Content Table 2, available at: http://links.lww.com/MS9/A963.

**HIGHLIGHTS**

- Self-medication means the usage of medication without the authorization or prescription from a certified physician. It is a rising concern, especially in middle and low-income countries. Several studies have reported data on self-medication practices, but studies reporting trends and issues regarding self-medication in the pediatric population are scarce.
- This cross-sectional study aims to determine the frequency of self-medication in children practiced by their caregivers, and factors leading to it. It recruited 476 children, whose caregivers went through an interviewer-administered structured questionnaire. Most of the children were males, with their ages mostly ranging from 1 to 3 years.
- The results showed that most of the caregivers were illiterate, and mostly the females did not have easy access to nearby healthcare facilities. Moreover, in the majority of cases, the nearest healthcare facility was located more than 5 km away from the children’s residency. Most households had low income, and they consisted of extended families. Presumption of illness not being very severe, which was fever in the majority, led to increased usage of medications at home. Irrational use of drugs was also widespread, with caregivers recommending and spreading the drugs to others, thus promoting this trend to other families.
- The research has also found that there is no significant gender difference in self-medication practices, but illiteracy is highly associated with self-medication. Moreover, taking care of caregiver children with self-medication was related to the age of children, the education of the caregiver, the income of the caregiver, family type, and severity of illness. Self-medication practice rate was found to be higher in children aged 1–3 years, illiterate caregivers, low household income, extended families, and in mild illnesses. Moreover, there is a shortage and inequalities in the geographic distribution of HCFs, with the HCF being over 5 km away for most of the population.
- Based on the results, the need of the hour is to increase the literacy rate and ease of access to healthcare facilities for the general population. Moreover, facilities should be widely accessible and affordable to all caregivers so that they don’t self-medicate their children based on previous experiences or general recommendations.

**Figure 2.**
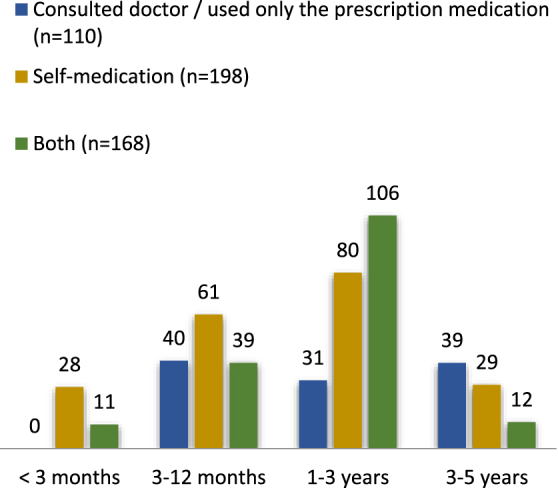
The method of treatment received by children in the last 3 months.

**Figure 3.**
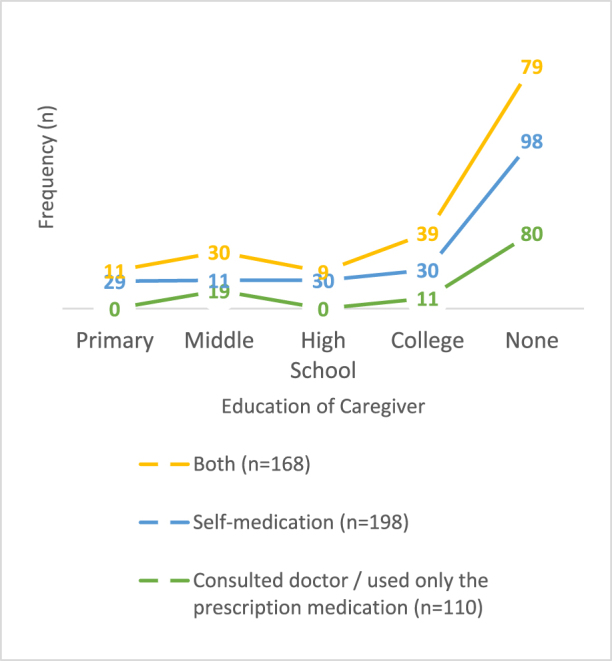
Illustrates the relation of the SM with education level.

As shown in Supplementary Digital Content Table 3, available at: http://links.lww.com/MS9/A964, significant associations could be observed between the method of treatment observed and the socio-demographics. Three-quarters of participants (*n* = 366/476 76.8%) reported having been using SM as a treatment option in the last 3 months, 198 (41.59%) of which did SM alone while 168 (35.2%) did SM along with consulting a doctor and that too majorly (36.9%) in the age group of 1–3 years (Fig. 2). On the other hand, 110 CGs solely consulted the doctor and used prescribed medication. There was not much difference observed in the correlation of SMC5 with gender, as the percentage of males and females being self-medicated was 39.7 and 44.0%, respectively. Furthermore, CGs with the least level of education were found to have a major (49.5%) share in the practice of SM (Fig. 3).

Out of the 198 CGs, 187 CGs who preferred only SM over consulting a doctor had a health facility nearby, 54% of which were civil dispensaries. But the same percentage of health facilities were without any authorized medical practitioner, and more than 5 km away. Moreover, a significant number of these CGs (177) reported the presence of a pharmacy nearby. The rest of the stratified methods of treatment according to access to healthcare are given in Supplementary Table 4, http://links.lww.com/MS9/A965.

Our study received 476 responses from direct CGs, of which 366 (76.89%) had SM within the past 3 months, approximately 4 times higher than the non-SM group. Children in the group 1–3 years have more than 50% SM while the non-SM group accounted for 28.18%, while children in the group of 3–5 years had the opposite trend, shown at the rate of 35.45% non-SM is 3 times higher than the SM group, the difference between the two groups is statistically significant (P < 0.001).

Table 1 also shows that there is a statistically significant difference in the SM group compared with the non-SM group, shown in variables including male children (58.74 vs 46.79%); male CG (100 vs 0%); the CG has a university degree (18.85 vs 10.00%); CG with income less than 10 000 (34.97 vs 3.64%); family with one or two children (46.45 vs 17.27%); the number of family members is less than or equal to 4 (13.38 vs 9.09%); extended family (65.30 vs 26.36%); CG is the housewife (78.42 vs 90.00%); health facility (86.61 vs 100.00%); pharmacy (83.88 vs 100.00%). Habits of people using SM for children in their care are given in Supplementary Digital Content Table 5, available at: http://links.lww.com/MS9/A966.

**Table 1:** Basic characteristics among the survey participants across self-medication usage status

Characteristics	Self-medication	Non-self-medication	[ALL]	N	*P*-value
	*N = 366*	*N = 110*	*N = 476*		
Age children				476	<0.001
< 12 months	139 (37.98%)	40 (36.36%)	179 (37.61%)		
1–3 years	186 (50.82%)	31 (28.18%)	217 (45.59%)		
3–5 years	41 (11.20%)	39 (35.45%)	80 (16.81%)		
Gender children				475	0.036
Female	151 (41.26%)	58 (53.21%)	209 (44.00%)		
Male	215 (58.74%)	51 (46.79%)	266 (56.00%)		
Caregiver relation				476	<0.001
Mother	347 (94.81%)	90 (81.82%)	437 (91.81%)		
Father	9 (2.46%)	0 (0.00%)	9 (1.89%)		
Grandmother	0 (0.00%)	20 (18.18%)	20 (4.20%)		
Grandfather	10 (2.73%)	0 (0.00%)	10 (2.10%)		
Age caregiver				476	<0.001
≤ 35 years	288 (78.69%)	50 (45.45%)	338 (71.01%)		
> 35 years	78 (21.31%)	60 (54.55%)	138 (28.99%)		
Education of caregiver				476	<0.001
University	69 (18.85%)	11 (10.00%)	80 (16.81%)		
Primary/mid/high school	120 (32.79%)	19 (17.27%)	139 (29.20%)		
None	177 (48.36%)	80 (72.73%)	257 (53.99%)		
Income				476	<0.001
<10,000	128 (34.97%)	4 (3.64%)	132 (27.73%)		
10 000–20 000	70 (19.13%)	10 (9.09%)	80 (16.81%)		
20 000–50 000	146 (39.89%)	76 (69.09%)	222 (46.64%)		
>50,000	22 (6.01%)	20 (18.18%)	42 (8.82%)		
Number children of family				476	<0.001
1	50 (13.66%)	0 (0.00%)	50 (10.50%)		
2	120 (32.79%)	19 (17.27%)	139 (29.20%)		
3	38 (10.38%)	0 (0.00%)	38 (7.98%)		
4	50 (13.66%)	10 (9.09%)	60 (12.61%)		
5	30 (8.20%)	10 (9.09%)	40 (8.40%)		
≥ 6	78 (21.31%)	71 (64.55%)	149 (31.30%)		
Number people of family				476	0.012
3	20 (5.46%)	0 (0.00%)	20 (4.20%)		
4	29 (7.92%)	10 (9.09%)	39 (8.19%)		
6	60 (16.39%)	10 (9.09%)	70 (14.71%)		
7	60 (16.39%)	19 (17.27%)	79 (16.60%)		
≥ 7	197 (53.83%)	71 (64.55%)	268 (56.30%)		
Family type				476	<0.001
Extended	239 (65.30%)	29 (26.36%)	268 (56.30%)		
Nuclear	127 (34.70%)	81 (73.64%)	208 (43.70%)		
Occupation of caregiver				476	0.004
Govt Employee	59 (16.12%)	11 (10.00%)	70 (14.71%)		
Housewife	287 (78.42%)	99 (90.00%)	386 (81.09%)		
Self-emp/Private business	20 (5.46%)	0 (0.00%)	20 (4.20%)		
Health facility				476	<0.001
No	49 (13.39%)	0 (0.00%)	49 (10.29%)		
Yes	317 (86.61%)	110 (100.00%)	427 (89.71%)		
HF type				439	0.492
Basic Health Unit	168 (51.06%)	61 (55.45%)	229 (52.16%)		
Civil dispensary	161 (48.94%)	49 (44.55%)	210 (47.84%)		
MP appointed				438	0.510
No	160 (48.78%)	49 (44.55%)	209 (47.72%)		
Yes	168 (51.22%)	61 (55.45%)	229 (52.28%)		
Pharmacy medical store				476	<0.001
No	59 (16.12%)	0 (0.00%)	59 (12.39%)		
Yes	307 (83.88%)	110 (100.00%)	417 (87.61%)		

Table 2 shows the value of univariable and multivariable logistic regression predicting SM in the past 3 months. The results show that taking care of children with SM was related to the age of the child, the education of the CG, the income of the CG, and family type. Accordingly, the multivariable model showed that the group of 1-3-year-olds had a 2.31 times higher probability of SM than the group of children under 12 months, but the group of 3–5-year-olds had this probability 96% lower than that of the group of children under 12 months. CG with university education tended to have SM 2.17 times higher than the other group; the results of the multivariate analysis were close to statistical significance (*P* = 0.073). An increase in income of CG was associated with a tendency to decrease in the rate of SM compared with the group with an income of less than 10 000, with a decrease of 98, 99, and 79% respectively; this difference was statistically significant (*P* < 0.05). The nuclear family type had a lower rate (89%) of SM than the extended family type. The parameters of the univariable model and multivariable model had no significant difference, and the adjusted R^2^ of the model was 37.4%.

**Table 2:** Multivariable logistic regression predicts self-medication for children

	**Univariable**			**Multivariable**		
Predictors	**OR**	**95% CI**	***P*-value**	**OR**	**95% CI**	***P*-value**
Age children						
< 12 months	*Reference*			*Reference*		
1–3 years	1.78	1.06–3.03	0.030	2.31	1.20–4.53	0.013
3–5 years	0.30	0.17–0.53	<0.001	0.04	0.01–0.11	<0.001
Education of caregiver						
No	*Reference*			*Reference*		
University	2.07	1.09–4.28	0.035	2.17	0.95–5.22	0.073
Income						
< 10,000	*Reference*			*Reference*		
10 000–20 000	0.22	0.06–0.68	0.013	0.02	0.00–0.08	<0.001
20 000–50 000	0.06	0.02–0.15	<0.001	0.01	0.00–0.03	<0.001
> 50,000	0.03	0.01–0.10	<0.001	0.21	0.05–0.74	0.020
Family type						
Extended	*Reference*			*Reference*		
Nuclear	0.19	0.12–0.31	<0.001	0.11	0.05–0.20	<0.001
Observations	475			475		
R^2^ Tjur				0.374		

**Figure 4.**
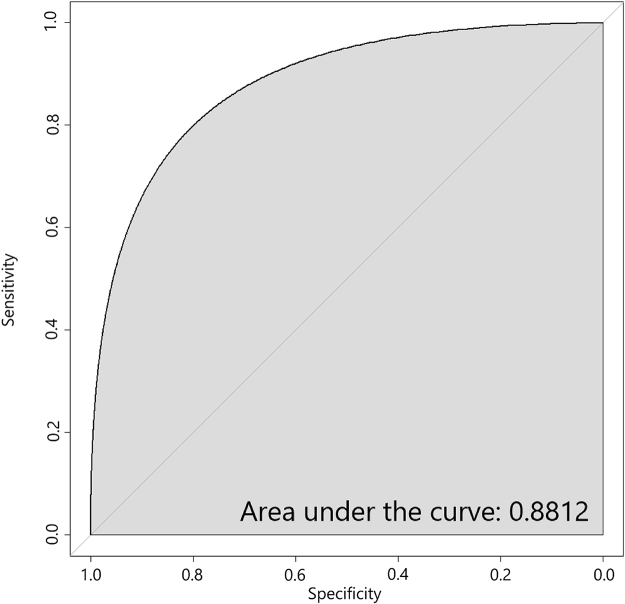
ROC curve for predicting self-medication.

Figure 4 shows the multivariable logistic regression predicting SM for children with variables including age of children, education of CG, income of CG, and family type. The model that fits our data, obtained with the area under the ROC curve, is 88.12%, with a sensitivity of 80%, and the specificity is also above 80%.

**Figure 5.**
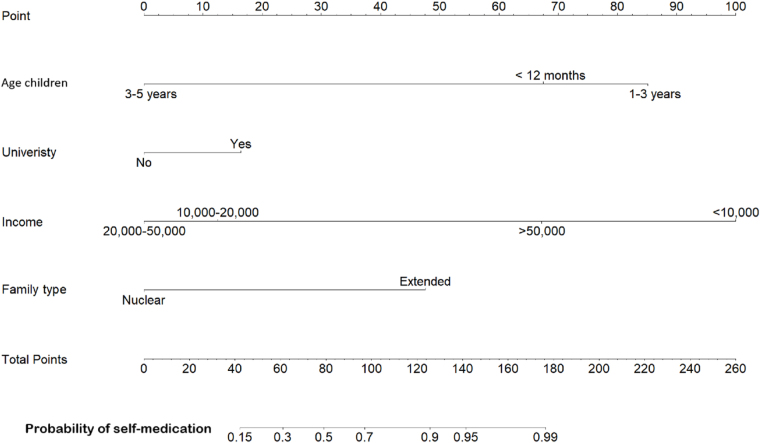
Nomogram predicting self-medication for children.

As depicted in Figure 5, the highest probability of SM is observed in the group with income less than 10 00 PKR, followed by the children of the 1–3 years of age group, with scores of 100 and 85 points, respectively. On the contrary, CGs with nuclear families and a level of education up to university have the least probability of SM.

**Figure 6.**
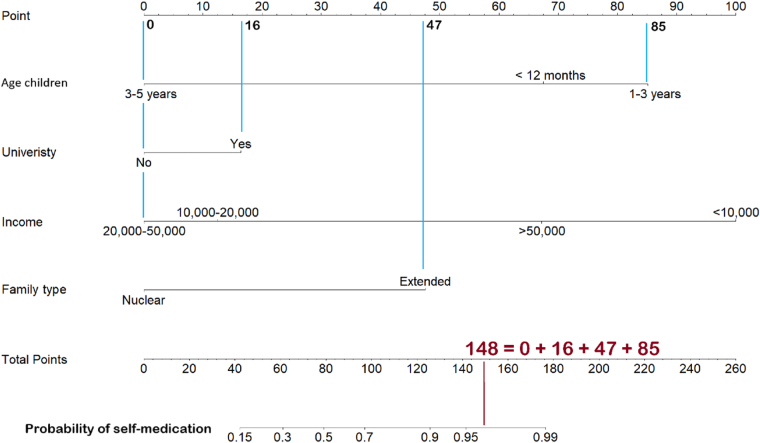
Predictive outcomes for self-medication in children.

Figure 6 shows the predictive outcomes for SM in children. Examples of cases where research subjects include respondents who are caring for children in the 1–3 age group (85 points), with a CG in the group with a university degree (16 points), the average income of this person is between 20 000 and 50 000 (0 points), and these children live in an extended family (47 points). Thus, the total score of this patient is 148 points, corresponding to the probability of using SM being over 95%. When compared with actual data, we found that 100% (60/60 study subjects had the characteristics outlined above) all use SM to treat their children’s illnesses.

## Discussion

Baluchistan is the largest province of Pakistan with regard to area. It is an underdeveloped region deprived of basic human needs, including health and education. Primary causes of high illiteracy rates include the absence of nearby high schools, local transport deficit, the unwillingness of parents to educate females, and poor financial status. The mountainous and rough terrain of the province is unfavorable for building schools, limiting the access of the local population to education and hindering the overall development and growth of the province^[16]^.

The use of SMC5 for mild illnesses is widespread. SMC5 is predominant for symptoms like fever, cough, diarrhea, eye diseases, and abdominal pain^[12,15]^. Lack of time, in the case of the working status of both parents, and easy access to the pharmacies, as well as easy availability of OTC drugs, make it more feasible for CGs to treat such mild symptoms at home. Therefore, CGs prefer to use the last filled prescription to save time and money^[3,5]^. However, it may lead to the underdiagnosis of the underlying disease, with the development of drug side effects.

As in Cameroon^[17]^, our study also found a disparity in the geographic distribution of HCFs, with the HCF being over 5 km away for most of the population, as shown in Fig. 5. Thereby, convincing CGs to prefer SM over visiting distant HCFs for a physician’s advice. Similar results were found in Chinese and Tanzanian studies^[18,19]^.

Worldwide, women play the role of a CG and men take up the responsibility of earning bread and butter for the family, conforming to cultural and social norms. In conservative societies, females also rely on males for their access to a pharmacy or a healthcare facility, as per the social norms^[1,5]^. Similar to a study conducted in Tanzania, our study found that 96% of the CGs were females^[19]^. However, we found no significant association between the frequency of SMC5 and the gender of the child, a finding consistent with the study conducted by Elong Ekambi *et al*^[20]^. Additionally, young marriages are very common in Pakistan; young CGs are mostly inexperienced and lack the proper parenthood skills, so it also contributes to the risk of SM^[21]^.

SMC5 is associated with a lack of a higher level of education^[1,22–24]^. The majority of CGs in our study had not even acquired primary-level education, especially females. However, this finding is not consistent with other studies conducted in Romania and Italy, which reported that higher education among CGs generated confidence to self-medicate their children^[25–27]^.

Our study reveals an inverse relationship between the income of the families and SMC5, unlike the study conducted by Ahmed *et al*^[27]^. which reports the prevalence of SMC5 in families with high income. This finding suggests that financial constraints also promote SM, keeping the CGs from high consultation charges. This is similar to the relationship found in the studies conducted in Cameroon^[20]^, China^[18]^, Mexico^[28]^, Indonesia^[27]^, and Colombia^[24]^.

As in the present study, the study conducted in Northern Uganda also found that SM predominates in extended families and the constant source of information for SM remains the family members (Fig. 4). Another potential reason for the circulation of medicines among the members of the family can be the experience of the drug, and the resemblance of the symptoms of illness in the past.

Children, being a vulnerable population, are inherently predisposed to adverse drug reactions^[29]^. Children do not have fully developed organs to allow proper metabolism, and the dose of medicine must be balanced with its therapeutic effect to avoid drug toxicity^[30]^. However, the majority of the CGs are unaware of the possible drug reactions and their adverse effects. Therefore, this knowledge gap must be filled to control the rise in the practice of SM^[31]^.

## Limitations

The findings of this cross-sectional study are not generalisable as this is a single-centre study that was conducted in a population with a very low literacy rate and limited healthcare facilities. Secondly, it does not represent the status and prevalence of SM among the adult population. Moreover, it also has potential implications for recall bias, as the responses from participants were subject to their ability to recall their use of medications at home.

## Conclusion

This study shows that SMC5 is a widespread phenomenon with notable prevalence among CGs with lower levels of education and income, suggesting a potential correlation between socioeconomic status and healthcare-seeking behavior. A substantial majority of CGs had poor knowledge regarding the appropriate usage of the drug, including dosage, timing, and potential side effects. Therefore, this rising issue should be addressed by the mutual efforts of healthcare organizations, educational institutions, and the government. The negative repercussions of the SMC5 should be stressed to the community via awareness programs and patient counseling, along with providing necessary support and resources.

## STROCCS criteria

The work has been reported in line with STROCCS criteria^[32]^.

## Data Availability

Not applicable.
